# Assessment of the appropriateness of cardiovascular preventive medication in older people: using the RAND/UCLA Appropriateness Method

**DOI:** 10.1186/s12877-022-03082-8

**Published:** 2022-05-05

**Authors:** Milly A. van der Ploeg, Rosalinde K. E. Poortvliet, Wilco P. Achterberg, Simon P. Mooijaart, Jacobijn Gussekloo, Yvonne M. Drewes

**Affiliations:** 1 Department of Public Health and Primary Care, Leiden University Medical Center, Leiden, the Netherlands; 2 Department of Internal Medicine, Section Gerontology and Geriatrics, Leiden University Medical Center, Leiden, the Netherlands

**Keywords:** Cardiovascular diseases, Clinical decision-making, Drug therapy, Geriatrics, Preventive medicine

## Abstract

**Background:**

In clinical practice and science, there is debate for which older adults the benefits of cardiovascular preventive medications (CPM) still outweigh the risks in older age. Therefore, we aimed to assess how various clinical characteristics influence the judgement of appropriateness of CPM in older adults.

**Method:**

We assessed the appropriateness of CPM for adults ≥75 years with regard to clinical characteristics (cardiovascular variables, complexity of health problems, age, side effects and life expectancy) using the RAND/ University of California at Los Angeles Appropriateness Method. A multidisciplinary panel, including 11 medical professionals and 3 older representatives of the target population, received an up-to-date overview of the literature. Using 9-point Likert scales (1 = extremely inappropriate; 9 = extremely appropriate), they assessed the appropriateness of starting and stopping cholesterol lowering medication, antihypertensives and platelet aggregation inhibitors, for various theoretical clinical scenarios. There were two rating rounds, with one face-to-face discussion in between. The overall appropriateness judgments were based on the median panel ratings of the second round and level of disagreement.

**Results:**

The panelists emphasized the importance of the individual context of the patient for appropriateness of CPM. They judged that in general, a history of atherosclerotic cardiovascular disease strongly adds to the appropriateness of CPM, while increasing complexity of health problems, presence of hindering or severe side effects, and life expectancy < 1 year all contribute to the inappropriateness of CPM. Age had only minor influence on the appropriateness judgments. The appropriateness judgments were different for the three types of CPM. The literature, time-to-benefit, remaining life expectancy, number needed to treat, and quality of life, were major themes in the panel discussions. The considerations to stop CPM were different from the considerations not to start CPM.

**Conclusion:**

Next to the patients’ individual context, which was considered decisive in the final decision to start or stop CPM, there were general trends of how clinical characteristics influenced the appropriateness, according to the multidisciplinary panel. The decision to stop, and not start CPM, appeared to be two distinct concepts. Results of this study may be used in efforts to support clinical decision making about CPM in older adults.

**Supplementary Information:**

The online version contains supplementary material available at 10.1186/s12877-022-03082-8.

## Background

World-wide, millions of older adults use cardiovascular preventive medication (CPM) to reduce their high risk of morbidity and mortality from atherosclerotic cardiovascular diseases (ASCVD). Numerous randomized controlled trials [1–4] have shown that up to high ages, this risk can be decreased effectively and safely by cholesterol lowering medication, antihypertensives, and platelet aggregation inhibitors (PAI).

Changes in physiology and clinical presentation due to ageing, may alter the balance between risks and benefits of CPM. Some changes, such as age- or disease related decline of organ functions, the co-existence of multiple morbidities, and polypharmacy increase the risk of medication related harms and burden [5–7]. Moreover, there are changes that can reduce the benefits of CPM, such as competing risks of death from non-ASCVD, or short life expectancy [8–10]. As a result, the balance between medication related risks and benefits may reach a point where the disadvantages of preventive medication no longer outweigh the potential advantages with sufficient margin; at this point medication has become inappropriate.

People of a very high age, with complex health problems, polypharmacy or with a short life expectancy are generally not well represented in randomized controlled trials [11, 12]. This has fueled the debate to which extent the supporting evidence can be generalized, and complicates clinical decision making [13–18].

We aimed to assess how various clinical characteristics, especially cardiovascular variables, age, life expectancy, complexity of health problems and medication related hindering side effects, influence the appropriateness of CPM in older adults.

## Methods

**Fig. 1:**
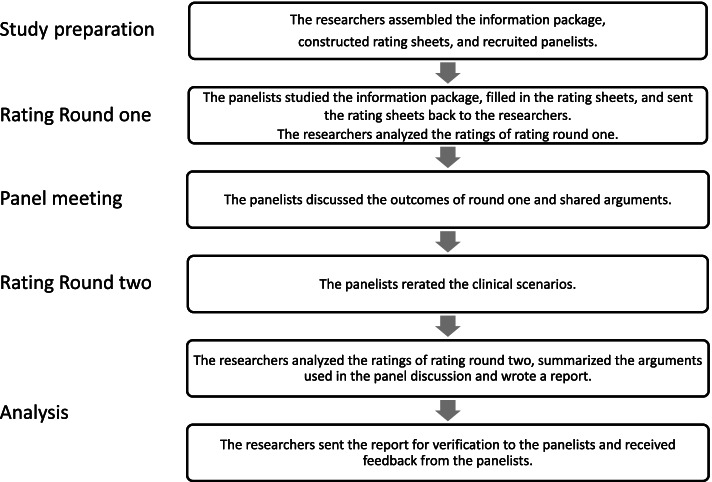
Flow chart of the study

We used the RAND/ University of California at Los Angeles Appropriateness Method (RAM). The RAM is a validated scientific method which integrates scientific evidence with the collective judgment of experts. After a preparatory phase, the panelists assessed the appropriateness of clinical scenarios individually at home. Thereafter a face-to-face panel discussion supervised by an independent chairman was organized followed by a second individual rating round [19]. See Fig. 1 for the study flow chart.

### Study preparation

#### Assembly of the information package

We provided all panelists with an extensive information package, including an overview of recent literature (for details see Additional file 1), recommendations by current international cardiovascular disease prevention guidelines, and a list of definitions used in the study.

#### Construction of the rating sheets and definitions

We constructed rating sheets to separately rate the appropriateness of starting and stopping cholesterol lowering medication, antihypertensives, or PAI. These rating sheets consisted of theoretical clinical scenarios constructed by combining clinical variables, and 9-point Likert scales. Based on a literature search, previous research [20, 21], clinical experience of the researchers, and clinical guidelines [22], we selected the following clinical variables that could possibly influence the appropriateness of starting and stopping CPM: cardiovascular variables, age, life expectancy, complexity of health problems and medication related hindering side effects. Cardiovascular variables included: history of ASCVD (< 1 year ago/ ≥1 year ago/no), low-density lipoprotein cholesterol (LDL-C) level (> 2.5 mmol/l) and systolic blood pressure (SBP) (140 mmHg/160 mmHg/180 mmHg). The appropriateness of stopping CPM was also assessed for SBP = 120 mmHg and LDL-C ≤ 2.5 mmol/l. The diastolic blood pressure was set at ≥70 mmHg in all clinical scenarios regarding antihypertensive treatment. Besides the cardiovascular variables, age (75–85/ > 85), life expectancy (< 1 year/≥1 year), and the complexity of health problems were included. Complexity of health problems was expressed as the number of health domains with problems (0/1/2/3–4). This definition was based on a previous study [23] that showed that in adults ≥75 years, besides the physiological (somatic) domain and the functional domain, two other domains (mental and social) are also related to poor health and wellbeing in older individuals. A health problem was defined as ‘a problem that causes the patient to experience a limitation in his/her daily functioning’. To assess the appropriateness of stopping in the presence of side effects, we included the variable ‘medication related hindering side effects’ (present/ absent). It was agreed that for these clinical scenarios the panelist would assume that different kinds of the relevant CPM have been tried, all resulting in hindering side effects. For PAI, a hindering side-effect was divided in mild and severe, the latest indicating the need for medical attention. Detailed information about the construction of the rating sheets, the categories of the clinical variables and an example of a rating sheet is provided in Additional file 2.

The panelists were instructed to consider CPM appropriate when the expected benefits exceed the negative consequences by a sufficiently wide margin that treatment is worth doing [19]. Stopping was defined as ‘the intention to discontinue medication permanently, with acceptance of increase in LDL-C level or SBP’. Switching medication was not considered as stopping.

#### Composition of the panel

The composition of the panel was based on a representation of the physicians prescribing CPM in older adults, supplemented with a pharmacist, a medical ethics expert and older representatives of the target population. The panelists were recruited by personal invitations sent by the researchers. We pursued diversity in scientific and clinical experience, age and gender, as well geographic spread throughout the Netherlands. Selection criteria were: affinity with the subject, willingness to invest the necessary time and effort to participate in the study (approximately 10 hours of preparation time and 2 days of panel meetings), and no conflicts of interest. The panel included 1 cardiologist, 1 clinical geriatrician, 3 general practitioners, 2 elderly care physicians, 1 internal medicine specialist, 1 neurologist, 1 medical ethics expert, 1 pharmacist and 3 older representatives of the target population (recruited from the Older Persons Advisory Board ‘Care and Wellbeing’ South-Holland North) [24]. The total panel consisted of 14 panelists (9 males/5 females), with an average of 24 years of clinical experience (range from 11 to 40 years). The panel was chaired by an experienced discussion leader.

### Rating process and panel meeting

Six weeks before the panel meeting the information package and rating sheets for round one were sent to the panelists. For each clinical scenario (*n* = 450 in total) the panelist rated the appropriateness of starting and stopping cholesterol lowering medication, antihypertensives and PAI on 1 to 9-point Likert scales (1 = extremely inappropriate; 9 = extremely appropriate). They were instructed to weigh the evidence and to use their expert opinion for the assessment of the appropriateness of starting or stopping CPM.

At the beginning of the panel meeting (June 2019) the results of the first round were reported back to the panelists on personalized forms, including his or her first-round rating, the panel median, and a frequency distribution. The individual ratings were blinded to the other panelists. The appropriateness judgments were based on the median panel rating and level of disagreement, using the following definitions: those with a median rating of 1 to 3, were classified as inappropriate, all scenarios with a median rating of 7 to 9, were classified as appropriate, the median rating of 3.5 to 6.5, were classified as uncertain. Disagreement was defined as: ‘for the same clinical scenarios, at least four panelists rated in the 1 to 3 range, and at least four panelists rated in the 7 to 9 range’. Results of round one merely serviced as discussion starters and were not part of the final appropriateness judgments of round two [19, 25].

The meeting started with a clarifying session in which the panelists discussed definitions. After the clarifying session the panelists discussed the appropriateness of starting and stopping of each CPM in context of the clinical scenarios. The panelists were invited by the chairman to share their arguments and perspectives, and were free to bring in additional literature. At the end of each discussion, they rerated the clinical scenarios (rating round two). Based on the outcomes of the first round, 24 extra clinical scenarios were added for the second round to evaluate the appropriateness of starting antihypertensive medication when SBP = 180 mmHg more in-depth, resulting in 474 clinical scenarios in total.

The entire discussion was audiotaped, and two researchers (YD, MP) made field notes. A report was written, and sent to the panelists for their comments.

### Analysis

The final appropriateness judgements for each scenario were based on the median panel rating and level of disagreement in the second round.

**Fig. 2:**
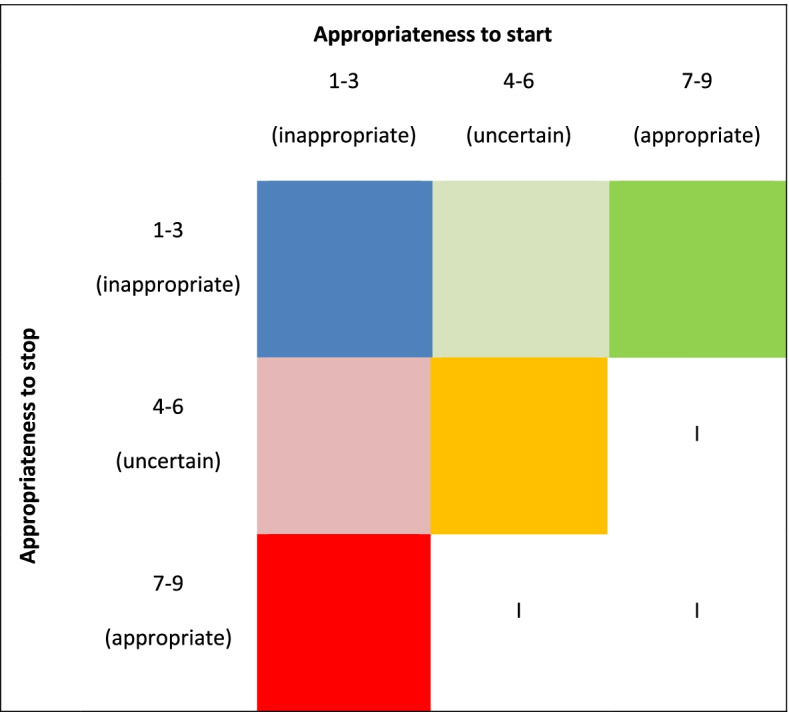
Combinations of the appropriateness judgments of starting and stopping medication. Legend: I = Inconsistent outcome: clinically impossible combination of appropriateness judgment of starting and stopping

We combined the appropriateness judgments for starting and stopping for each corresponding clinical scenario. Figure 2 shows all combinations of the appropriateness judgements for starting and stopping, as well as the color-coding used in the figures. Based on this color-coding the impact of cardiovascular variables, age, life expectancy, complexity of health problems, and hindering side effects on the appropriateness of prescribing CPM is visualized. In addition, to explain these findings, we analysed the report of the entire panel discussion and described their arguments.

## Results

One panelist missed the deadline for completing the ratings of round one, and one panelist had to leave early in round two. The rating sheets of the other panelists were 100% complete.

Outcomes of the appropriate judgements about starting and stopping CPM did not differ a lot between the two rounds. It appeared that after the panel discussion, the uncertainty was slightly decreased resulting in a slightly increased amount of ‘inappropriate’ judgements. The amount of scenarios which were assessed as ‘appropriate’ was almost the same in both rounds.

### Detailed figures with outcomes of ratings

**Fig. 3:**
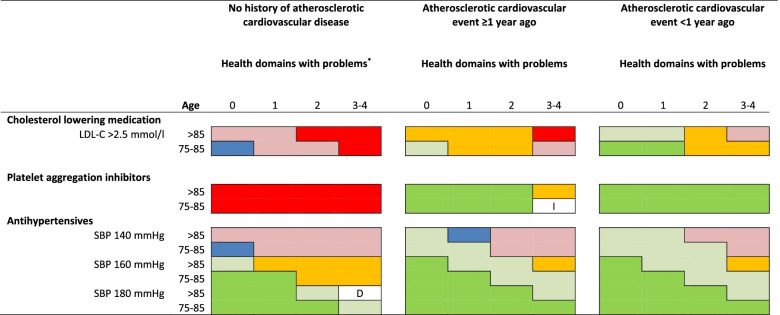
Appropriateness of cardiovascular preventive medication in patients ≥75 years. ^*^Number of health domains (somatic, functional, mental, social) with problems limiting daily functioning, range 0–4. Note: a) diastolic blood pressure was set to ≥70 mmHg; b) the appropriateness judgments displayed in this figure and Fig. 5 are combinations of the appropriateness judgments for starting and stopping cardiovascular preventive medication. Abbreviations: D=Disagreement: at least four panelists rated in the 1–3 range and at least four panelists rated in the 7–9 range; I = Inconsistent outcome: clinically impossible combination of appropriateness judgment of starting and stopping; LDL-C=Low-density lipoprotein cholesterol; SBP = systolic blood pressure

**Fig. 4:**
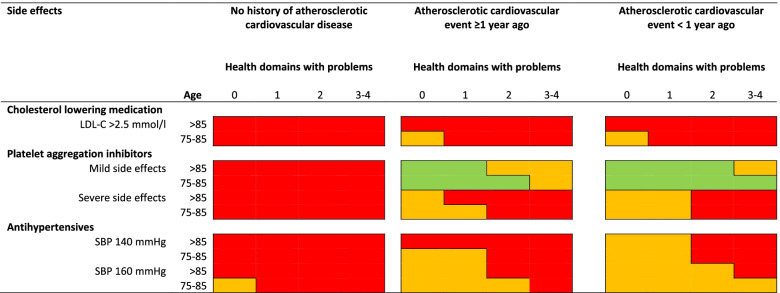
Appropriateness of cardiovascular preventive medication in patients of ≥75 years, in presence of side effects. Number of health domains (somatic, functional, mental, social) with problems limiting daily functioning, range 0–4. Note: diastolic blood pressure was set to ≥70 mmHg. Abbreviations: LDL-C= Low-density lipoprotein cholesterol; SBP = systolic blood pressure

**Fig. 5:**
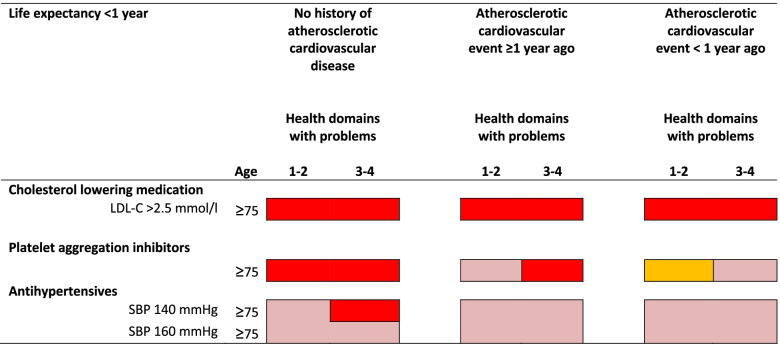
Appropriateness of cardiovascular preventive medication in patients of ≥75 years, when life expectancy < 1 year. Number of health domains (somatic, functional, mental, social) with problems limiting daily functioning, range 0–4. Note: a) diastolic blood pressure was set to ≥70 mmHg; b) the appropriateness judgments displayed in this figure and Fig. 3 are combinations of the appropriateness judgments for starting and stopping cardiovascular preventive medication. Abbreviations: LDL-C= Low-density lipoprotein cholesterol; SBP = systolic blood pressure

We summarized the main results into three figures (Figs. 3, 4 and 5). In these figures the outcomes of the combined appropriateness judgment for starting and stopping medication are displayed, using the colours as described in Fig. 2. See Additional file 4 for all appropriateness scores. In general, these figures show the impact of cardiovascular variables, age, life expectancy, complexity of health problems, and hindering side effects on the appropriateness of prescribing CPM. As visualised in Fig. 3, the appropriateness of prescribing PAI was mostly influenced by the history of ASCVD, and almost not depending on age and complexity of health problems. For antihypertensive and cholesterol lowering medication, the figure shows that, besides the effect of SBP-level on prescription of antihypertensive treatment, increasing complexity of health problems, and to a lesser extent increasing age, negatively influence the appropriateness of prescribing CPM. Figure 4 shows that hindering side effects of CPM were almost never accepted in patients without a history of ASCVD, leading to the judgement that CPM is inappropriate for this group. In patients with a history of ASCVD, the appropriateness of prescribing CPM depends on the seriousness of the side effects in case of PAI, and is negatively influenced by increasing age and increasing complexity of health problems in case of antihypertensive and cholesterol lowering medication. For patients with a low life expectancy, prescription of CPM was judged not being appropriate anymore (Fig. 5).

### Main patterns and arguments

#### Individual context

During the clarifying session it was discussed that in clinical practice the decision on starting or stopping CPM is based on two elements: the scientific evidence (population level) and the individual clinical context of a patient (individual level). The panelists agreed to focus the discussions and ratings on the level of scientific evidence as much as possible. However, they emphasized that in practice, the individual context of the patient is leading. Therefore, the panelists noted that the individual context of a patient may alter the appropriateness of starting or stopping, in a way that the final clinical decision may be different than the panels’ judgement.

#### Complexity of health problems

There was a general trend that, with increasing complexity of health problems, the combined appropriateness judgments for CPM tended to shift from appropriate to start and inappropriate to stop, through uncertain to start and uncertain to stop, towards inappropriate to start and appropriate to stop (Fig. 3). Although less pronounced, a similar trend was seen for increasing age. However, complexity of health problems had little influence on the appropriateness judgements of PAI in absence of hindering side effects.

The panelists reasoned that with increasing age, and accumulating health problems, older adults increasingly deviate from the average trial participant. Therefore, they especially questioned the generalizability of trials results for older adults with complex health problems. Regarding antihypertensives it was mentioned, that in general, for older adults the risk to develop side effects is higher, and this risk is often related to treatment intensity. Also, side effects are more likely to be more severe in older adults, especially when they are less healthy. This resulted in a trend to consider strict blood pressure regulation less appropriate with increase of complexity of health problems and age (Figs. 3, 4 and 5). In the discussions about cholesterol medication and PAI similar arguments were mentioned.

Another argument was that time to benefit can conflict with remaining life expectancy. The panelists reasoned that complex health problems are related with shortened life expectancy, and they considered the time-to-benefit of cholesterol lowering medication relatively long. As results the panelists judged that for older adults with complex health problems, it was inappropriate to start, and uncertain or appropriate to stop cholesterol lowering medication (Fig. 3). In contrast, in clinical scenarios with ASCVD, the time to benefit of PAI was considered relatively short. For these scenarios the panelists judged that, regardless of complexity of health problems, starting PAI was appropriate, and stopping PAI was inappropriate (Fig. 3).

#### History of ASCVD

The panelists argued that in old age, and in presence of complex health problems, a previous ASCVD event remains a strong risk factor for future ASCVD events. They expected most benefit for those with a history of ASCVD and noted that the number needed to treat is lower after an ASCVD event. This led the panelists to judge that for most clinical scenarios with ASCVD it was appropriate or uncertain to start, and inappropriate to stop CPM (Fig. 3). Regarding antihypertensive treatment, the panelists tended to target at lower SBP value for people with higher ASCVD risk, especially for older adults without complex health problems. When there was no previous ASCVD, the panelists more often judged starting inappropriate, and stopping uncertain or appropriate. This trend was most clear for PAI, and a similar trend was seen for cholesterol lowering medication (see Additional file 3 for the considerations). Last, there was disagreement about the appropriateness of stopping cholesterol lowering medication when LDL-C level ≤ 2.5 mmol/l in relatively young and healthy older adults without ASCVD; some panelists considered low LDL-C on treatment prove of effective treatment and, a reason to continue, while others considered it an extra reason to stop (Additional file 4: Table 3.1).

#### Side effects, life expectancy and quality of life

Severe or hindering side effects and a life expectancy < 1 year, were both strong reasons to shift the judgement of CPM towards inappropriate to start/ appropriate to stop (Figs. 4 and 5). The most important argument for this trend was that these side effects have a negative impact on quality of life and/or daily functioning. The panelists discussed that in general, quality of life becomes more in focus when health deteriorates and the end of life approaches. Consequently, it was generally considered appropriate to stop CPM in order to improve the quality of life or daily functioning in presence of complex health problems, and/or short life expectancy, especially when the ASCVD-risk was relatively low, or when the risk of death by non-ASCVD was high. However, it was noted that when the risk of ASCVD is high and a person is relatively young and healthy, the benefits of treatment may outweigh the burden, and it may appropriate to continue treatment despite the presence of hindering side effects. As a consequence, some clinical scenarios with side effects were judged uncertain (Fig. 4). The panelists were more reluctant towards the appropriateness of stopping PAI in presence of ASCVD, especially when side effects were mild (Fig. 4). See Additional file 3 for more discussion about this topic.

#### ‘Not starting’ and ‘stopping’: two different concepts

The panelists concluded that the decision not to start medication is different from the decision to stop medication. They were generally more reluctant and uncertain about stopping compared to ‘not starting’. It was reasoned that for an 85 year old individual, evidence to newly start CPM is rather weak. However, if this person already has been using CPM since the age of 70, they were reluctant to stop because evidence for starting medication in the past was strong, and he endured it for 15 years already. Besides, high quality evidence about the safety of stopping is scarce, and stopping could be harmful. It was also mentioned that at the start of treatment it is unknown who will develop side effects, while for current users (in most cases) it is. An older representative mentioned that getting the advice to stop one’s medication can be perceived as ‘you have been given up’, and added that taking preventive medication can provide a sense of security. At the same time physicians expressed their reservations to ‘take away’ patients’ medication, and because of anticipated regrets if an ASCVD-event should occur following an advice to stop CPM.

For some clinical scenarios it was judged that it was appropriate not to change (the blue boxes in Fig. 3); on the one hand starting medication could lead to side effects (which can lower quality of life, even when unrecognized), and on the other hand, there was a discussion that stopping medication might disturb an internal balance. This sentiment was also underlined by older representatives of the target population. They added that knowing what you have is sometimes preferred over not knowing what you’ll get.

## Discussion

In this RAM study a multidisciplinary expert panel assessed the appropriateness of cholesterol lowering medication, antihypertensives and PAI, for older adults (≥75 years) in different clinical scenarios.

In addition to the panelists’ notion that the individual context is very important in the final decision to start or stop CPM, there were general trends in the appropriateness judgments. According to the panelists, history of ASCVD strongly added to the appropriateness of CPM, while increasing complexity of health problems, presence of hindering or severe side effects, and life expectancy < 1 year all contributed to the inappropriateness of CPM. Age had only minor influence on the appropriateness. The extent to which these factors influenced the appropriateness ratings depended on the type of CPM. In addition, the panelists discussed that there are different considerations when deciding not to start or to stop CPM, and sometimes it is appropriate not to change.

### Comparison with ASCVD prevention guidelines

When we compare the appropriateness judgments in this study with several influential international ASCVD prevention guidelines (hereafter ‘guidelines’), we noticed several differences [26–30]. First, the panelist often judged that the appropriateness of CPM is different for those with complex health problems compared to those without. In absence of complex health problems, the appropriateness ratings were mostly in concordance with the guidelines, especially for the clinical scenarios with previous ASCVD. For older adults with complex health problems, the panelists expressed more reluctance towards CPM. Although guidelines suggest to take factors into account that may change the risks and benefits of CPM in older adults, most do not provide separate recommendations regarding CPM for older adults with complex health problems, or with other health related issues like frailty or short life expectancy [13]. Second, results of this study show that considerations are different when deciding on not starting or stopping CPM. The difference between not starting and stopping is not clearly addressed by the guidelines. Third, the panelists strongly agreed that in presence of hindering side effects it is appropriate to stop CPM. In most guidelines recommendations on stopping CPM are limited to the safety aspects, only few mention stopping in context of age, quality of life or health problems [13, 26].

### Strengths and limitations

By using the RAM-procedure, we combined scientific evidence, clinical experience and client perspectives, to address a complex clinical dilemma. A strength of this study is that the results are not limited to the appropriateness judgments, but also contain arguments and considerations that add nuance to the debate. Another strength of this study is that it was innovative to include older representatives of the target population next to medical professionals in a multidisciplinary panel. Herewith the panel reflected the variety of specialties and older people involved in decisions on treatment.

It could be seen as a limitation that the older representatives did not cover the full diversity of the target population, and that we only invited Dutch experts. To maximize the generalisability, we selected experts from throughout the Netherlands, of various ages, clinical disciplines and expertise, and instructed them to focus on the literature instead of the individual context. Moreover, compared to other countries, Dutch doctors may be more reluctant to prescribe CPM to older adults with complex health problems. Results from case-vignette studies showed considerable cross-country variation in general practitioners’ advice on cholesterol lowering and antihypertensive treatment of (frail) older adults [20, 21]. It would be interesting if panels in other countries replicate this study to compare the findings. Finally, because daily practice is even more complex than our theoretical scenarios, we had to make a selection of most relevant variables and simplifications had to be made. The panelists were clearly aware of this limitation and emphasized that the individual context of a patient may alter the appropriateness of starting or stopping, in a way that the final clinical decision may be different from the panels’ judgment.

### Implications of the results and future research

The result of this RAM is a weighing of the scientific literature by experts which will contribute to evidence-based treatment decisions regarding CPM in older adults. In the communication with patients this knowledge will be discussed and combined with the individual context of a patient. Our results could direct guideline committees, and may be used to develop decision tools. We recommend that in efforts to improve the decision making about CPM, questions about complexity of health problems, ASCVD history, side effects, remaining life expectancy, and quality of life, are explicitly discussed in the decision-making process.

For future research, we revealed two interesting topics. First, we noticed that the current focus of deprescribing research is on barriers and enablers of stopping medication. However, studies on the decision not to start medication are needed. Second, the finding that for older adults it may sometimes be preferred not to change medication (not to start or to stop) to maintain the sense of internal balance, should be further investigated.

## Conclusion

According to the multidisciplinary panel, the patients’ individual context was considered decisive in the final decision to start or stop CPM. In addition, there were general trends of how the clinical characteristics influenced their appropriateness judgments. The scientific literature, quality of life, time-to-benefit, number needed to treat, and remaining life expectancy were major themes in the panel’s argumentation. The decision to stop, and not start CPM, appeared to be two distinct concepts. Results of this study may be used in efforts to support clinical decision making about CPM in older adults.

## Supplementary Information

**Additional file 1.** Full search strategy and references for the information package. This file includes the elements of the literature search strategy that was performed to provide the panelist with an overview of the latest relevant literature, and the resulting list of references that were included in the information package.

**Additional file 2.** Construction of the rating sheets: detailed information. This file includes a detailed description of how the rating sheets were constructed, and an example of a rating sheet.

**Additional file 3.** Additional considerations mentioned in the discussions. This text file contains additional considerations that were discussed regarding the appropriateness of platelet aggregation inhibitors and cholesterol lowering medication in older adults.

**Additional file 4.** Appropriateness scores of starting and stopping cardiovascular preventive medication. This file includes twelve tables (3.1–3.12) that display the appropriateness scores (panel medians) of rating round 2.

## Data Availability

Not applicable.

## Abbreviations

ASCVD: Atherosclerotic cardiovascular diseases
CPM: Cardiovascular preventive medication
D: Disagreement
I: Inconsistent
LDL-C: Low-density lipoprotein cholesterol
PAI: Platelet aggregation inhibitors
SBP: Systolic Blood Pressure
RAM: RAND/ University of California at Los Angeles Appropriateness Method
UCLA: University of California at Los Angeles
