# Supplementary material for: Assessment of the appropriateness of cardiovascular preventive medication in older people: using the RAND/UCLA Appropriateness Method
Source: BMC Geriatr. 2022 May 5;22:394. doi: 10.1186/s12877-022-03082-8 (PMC9069851; doi:10.1186/s12877-022-03082-8)
Supplement: Supplementary file 1 — Additional file 1. Full search strategy and references for the information package. This file includes the elements of the literature search strategy that was performed to provide the panelist with an overview of the latest relevant literature, and the resulting list of references that were included in the information package. [file 12877_2022_3082_MOESM1_ESM.doc]

# Additional file 1

# Full search strategy and references for the information package

The literature search was performed in Pubmed. Access dates were March 22 and 28, 2019.The search strategy comprised of several elements which are listed in Table 1.

The literature search was based on the systematic review that was performed in March 2016, for the Dutch multidisciplinary guideline for the prevention of cardiovascular disease [1]. The guidelines developers performed a systematic quality assessment of the literature that was selected for the guideline. They used validated Risk of Bias tables based on systematic assessment of predefined methodological quality criteria. We extended the original search by adding an extra query, and specifically searched for articles about cardiovascular preventive medication in palliative care and/or the end of life (published in the last 5 years).

The PubMed search resulted in 1243 unique articles that were screened for relevance based on titles. Selection was based on relevance for the present study; articles with as topic cardiovascular prevention with medication in older adults, published in English or Dutch. After the first selection 135 articles were left to screen by abstracts. During the selection process we also found 13 relevant articles by screening the reference lists of the selected articles. The final selection consisted of 65 articles, of which 41 were classified as ‘recommended literature’, and 24 as ‘optional literature’. There was no additional quality check for the selected studies. Both reference lists can be found at the end of this document. Also, the Dutch multidisciplinary guideline for the prevention of cardiovascular disease and it’s references were recommended literature [1].

A team of general practitioners, elderly care physicians and internal medicine specialist reviewed the information package for completeness.

**Reference**

1. Dutch College of General Practitioners; Cardiovascular Risk Managment (CVRM) 2019. Available from: [https://richtlijnendatabase.nl/richtlijn/cardiovasculair_risicomanagement_cvrm/inleiding_kwetsbaarheid.html#tab-content-accountability](https://richtlijnendatabase.nl/richtlijn/cardiovasculair_risicomanagement_cvrm/inleiding_kwetsbaarheid.html#tab-content-accountability) accessed 05-05-2020 2019.

Table 1

|  | Elements of the search |
| --- | --- |
| 1 | (("Hypertension"[mesh] OR hypertensi*[tiab] OR "blood pressure"[tiab] OR hbp[tiab] OR "Blood Pressure"[mesh]) AND ("Antihypertensive Agents"[mesh] OR "Antihypertensive Agents"[pharmacological action] OR antihypertensi*[tiab]) |
| 2 | (("antihypertensive agents"[mesh] OR "calcium channel blockers"[mesh] OR "Angiotensin-Converting Enzyme Inhibitors"[mesh] OR "Angiotensin Receptor Antagonists"[mesh] OR "Thiazides"[mesh] OR "Adrenergic beta-Antagonists"[mesh] OR "antihypertensive agents"[pharmacological action] OR "calcium channel blockers"[pharmacological action] OR "Angiotensin-Converting Enzyme Inhibitors"[pharmacological action] OR "Angiotensin Receptor Antagonists"[pharmacological action] OR "Adrenergic beta-Antagonists"[pharmacological action] OR antihypertensi*[tiab] OR "Calcium channel blockers"[tiab] OR "Calcium channel blocker"[tiab] OR "ACE-inhibitors"[tiab] OR "angiotensin-receptor blocker"[tiab] OR "angiotensin-receptor blockers"[tiab] OR "thiazides"[tiab] OR beta-blocker*[tiab] OR "Hypertension/drug therapy"[mesh]) |
| 3 | ("Reference Values"[majr] OR target*[tiab] OR threshold[tiab] OR reference value*[tiab] OR goal*[ti] OR target*[ti] OR reference value*[ti] OR intensi*[tiab] OR ((pressure[tiab] OR BP[tiab]) AND goal*[tiab]) OR elderly[ti] OR "very old"[ti] OR "oldest old"[ti] OR "80 years"[ti]) |
| 4 | (("Dyslipidemias"[mesh] OR "Hydroxymethylglutaryl-CoA Reductase Inhibitors"[mesh] OR "Simvastatin"[mesh] OR "Pravastatin"[mesh] OR "Rosuvastatin Calcium"[mesh] OR "Lovastatin"[mesh] OR "Atorvastatin"[mesh] OR statin*[ti] OR simvastatin*[tiab] OR pravastatin*[tiab] OR rosuvastatin*[tiab] OR lovastatin*[tiab] OR fluvastatin*[tiab] OR cerivastatin*[tiab] OR pitavastatin*[tiab] OR atorvastatin*[tiab]) |
| 5 | (("cardiovascular diseases"[mesh] OR "heart diseases"[mesh] OR "vascular diseases"[mesh] OR (("cerebrovascular"[tiab] OR "cardiovascular"[tiab] OR "heart"[tiab] OR "vascular"[tiab]) AND (disease*[tiab] OR disorder*[tiab] OR event*[tiab])) OR (myocard*[tiab] AND infarct*[tiab]) OR "cerebral vascular accident"[tiab] OR "CVA"[tiab] OR "stroke"[tiab] OR "TIA"[tiab]) AND ("Platelet Aggregation Inhibitors"[mesh] OR "Platelet Aggregation Inhibitors"[pharmacological action] OR "Aspirin"[mesh] OR "Dipyridamole"[mesh] OR "Prasugrel Hydrochloride"[mesh] OR "dipyridamol"[tiab] OR "clopidogrel"[tiab] OR aspirin*[tiab] OR "prasugrel"[tiab] OR "ticagrelor"[tiab] OR acetylsalicylic acid*[tiab] OR "Platelet Aggregation Inhibitor"[tiab] OR "Platelet Aggregation Inhibitors"[tiab]) AND ("primary prevention"[mesh] OR "secondary prevention"[mesh] OR (("primary"[tiab] OR "secondary") AND prevention[tiab]) OR prophyla*[tiab] |
| 6 | ("Aged"[mesh] OR aged[ti] OR "Frail Elderly"[mesh] OR "Aged, 80 and over"[mesh] OR "Aging"[mesh] OR "Geriatric Assessment"[mesh] OR "elderly"[tiab] OR "geriatric"[tiab] OR "community dwelling"[tiab] OR frail*[tiab] OR "aging"[tiab] OR "ageing"[tiab] OR septuagenarian*[tiab] OR octogenarian*[tiab] OR nonagenarian*[tiab] OR centenarian*[tiab] OR "old people"[tiab] OR "eldest"[tiab] OR "oldest"[tiab] OR "biological age"[tiab]) |
| 7 | ("meta-analysis"[ptyp] OR "meta-analysis as topic"[mesh] OR "meta analysis"[tw] OR "metanalysis"[tw] OR systematic review*[tw] OR literature review*[tw] OR "systematic"[sb] OR systematic overview*[tw] OR "Review Literature as Topic"[mesh] OR "cochrane"[tw] OR "cochrane"[all fields] OR "embase"[tw] OR "pubmed"[tw] OR "medline"[tw] OR "psychlit"[tw] OR "psyclit"[tw] OR "cinahl"[tw] OR "cinhal"[tw] OR "cancerlit"[tw] OR (("selection criteria"[tw] OR "data extraction"[tw]) AND "review"[ptyp]))) |
| 8 | ("clinical trial"[ptyp] OR "randomized controlled trial"[ptyp] OR "clinical trials as topic"[mesh] OR "randomized controlled trials as topic"[mesh] OR "Random Allocation"[mesh] OR "Double-Blind Method"[mesh] OR "Single-Blind Method"[mesh] OR "clinical trial, phase i"[ptyp] OR "clinical trial, phase ii"[ptyp] OR "clinical trial, phase iii"[ptyp] OR "clinical trial, phase iv"[ptyp] OR "controlled clinical trial"[ptyp] OR "randomized controlled trial"[ptyp] OR "multicenter study"[ptyp] OR "clinical trial"[ptyp] OR random*[tiab] OR ((clinic*[tw] OR clinical*[tw]) AND trial*[tw]) OR ((singl*[tw] OR doubl*[tw] OR treb*[tw] OR tripl*[tw]) AND (blind*[tw] OR mask*[tw])) OR "Placebos"[mesh] OR placebo*[tw] OR "RCT"[tw] OR random*[tw])) |
| 9 | ("Epidemiologic studies"[mesh] OR "case control studies"[mesh] OR "cohort studies"[mesh] OR "Controlled Before-After Studies"[mesh] OR "Case control"[tw] OR ("cohort"[tw] AND ("study"[tw] OR "studies"[tw])) OR cohort analy*[tw] OR ("follow-up"[tw] AND ("study"[tw] OR "studies"[tw])) OR ("observational"[tw] AND ("study"[tw] OR "studies"[tw])) OR "longitudinal"[tw] OR retrospective*[tw] OR prospective*[tw] OR consecutive*[tw] OR "cross sectional"[tw] OR "Cross-sectional studies"[mesh] OR "Historically Controlled Study"[mesh] OR "Interrupted Time Series Analysis"[mesh])) |
| 10 | ("Withholding Treatment"[mesh] OR "Deprescriptions"[Mesh] OR "cessation"[tw] OR stop*[tw] OR terminat*[tw] OR "cease"[tw] OR "ceasing"[tw] OR ceas*[tw] OR discontinu*[tw] OR "withdrawal"[tw] OR deprescr*[tw])) |
| 11 | Palliative Care/methods*[mesh] OR ”Palliative Care Medicine”[Mesh] OR ”Medicine, Palliative”[Mesh] OR ”Medicine, Palliative”[Mesh] OR ”Life Expectancy”[mesh] OR ”Cognition”[Mesh] OR ”Disorders*/complications”[Mesh] OR ”Cognition Disorders*/diagnosis” OR ”Cognition Disorders*/physiopathology” OR ”Cognition Disorders*/therapy” |
|  | Filters:  Language: Dutch or English  Publication date: since 2016/04/01, searches including element 11: since 2014/01/01  Publication type: not comment, not editorial  Species: humans |

Total hits: n=1253

🡪n=110 duplicates removed

n=1143 screened by title

🡪 n=1008 removed

n=135 selected based on title

🡪 n=83 removed

n=52 selected based on title & abstract (with second reviewer)

+ 13 titles included by snowballing

- n=41 recommended literature
- n=24 optional literature

# Reference list of Selected literature

## Recommended literature

1. Ridker PM, Lonn E, Paynter NP, Glynn R, Yusuf S. Primary Prevention With Statin Therapy in the Elderly: New Meta-Analyses From the Contemporary JUPITER and HOPE-3 Randomized Trials. Circulation 2017;135(20): p. 1979-1981.

2. Barter, P.J. and D.D. Waters, Variations in time to benefit among clinical trials of cholesterol-lowering drugs. J Clin Lipidol 2018;12(4): 857-862.

3. Ruscica M, Macchi C, Pavanello C, Corsini A, Sahebkar A, Sirtori CR. Appropriateness of statin prescription in the elderly. Eur J Intern Med 2018;50:33-40.

4. Bezin J, Moore N, Mansiaux Y, Steg PG, Pariente A. Real-Life Benefits of Statins for Cardiovascular Prevention in Elderly Subjects: A Population-Based Cohort Study. Am J Med. 2019;132(6):740-748.e7.

5. Korhonen MJ, Ilomäki J, Sluggett JK, et al. Selective prescribing of statins and the risk of mortality, hospitalizations, and falls in aged care services. J Clin Lipidol 2018; 12(3):652-661.

6. Kim K, Lee CJ, Shim CY, et al. Statin and clinical outcomes of primary prevention in individuals aged >75years: The SCOPE-75 study. Atherosclerosis 2019;284:31-36.

7. Corrao G, Monzio Compagnoni M, et al. Good adherence to therapy with statins reduces the risk of adverse clinical outcomes even among very elderly. Evidence from an Italian real-life investigation. Eur J Intern Med 2018;47:25-31.

8. Pilotto A, Gallina P, Panza F et al. Relation of Statin Use and Mortality in Community-Dwelling Frail Older Patients With Coronary Artery Disease. Am J Cardiol 2016;118(11):1624-30.

9. Kutner JS, Blatchford PJ, Taylor DH Jr, et al. Safety and benefit of discontinuing statin therapy in the setting of advanced, life-limiting illness: a randomized clinical trial. JAMA Intern Med, 2015. 175(5): p. 691-700.

10. Nishtala PS, Gnjidic D, Chyou T, Hilmer SN. Discontinuation of statins in a population of older New Zealanders with limited life expectancy. Intern Med J 2016;46(4):493-96.

11. Tjia J, Kutner JS, Ritchie CS, et al. Perceptions of Statin Discontinuation among Patients with Life-Limiting Illness. J Palliat Med 2017;20(10):1098-1103.

12. erlowitz DR, Foy CG, Kazis LE, et al. Effect of Intensive Blood-Pressure Treatment on Patient-Reported Outcomes. N Engl J Med 2017;377(8):733-44.

13. Odden MC, Peralta CA, Berlowitz DR, et al. Effect of Intensive Blood Pressure Control on Gait Speed and Mobility Limitation in Adults 75 Years or Older: A Randomized Clinical Trial. JAMA Intern Med 2017;177(4):500-7.

14. Supiano, MA, Williamson JD. Applying the Systolic Blood Pressure Intervention Trial Results to Older Adults. J Am Geriatr Soc 2017;65(1):16-21.

15. SPRINT MIND Investigators for the SPRINT Research Group, Williamson JD, Pajewski NM, et al. Effect of Intensive vs Standard Blood Pressure Control on Probable Dementia: A Randomized Clinical Trial. JAMA 2019;321(6):553-61.

16. Huang CJ, Chiang CE, Williams B, et al. Effect Modification by Age on the Benefit or Harm of Antihypertensive Treatment for Elderly Hypertensives: A Systematic Review and Meta-analysis. Am J Hypertens 2019;32(2):163-74.

17. Garrison SR, Kolber MR, Korownyk CS, McCracken RK, Heran BS, Allan GM. Blood pressure targets for hypertension in older adults. Cochrane Database Syst Rev 2017;8:Cd011575.

18. Corrao G, Rea F, Monzio Compagnoni M, Merlino L, Mancia G. Protective effects of antihypertensive treatment in patients aged 85 years or older. J Hypertens 2017;5(7):1432-41.

19. Sugawara M, Goto Y, Yamazaki T, et al. Low-Dose Aspirin for Primary Prevention of Cardiovascular Events in Elderly Japanese Patients with Atherosclerotic Risk Factors: Subanalysis of a Randomized Clinical Trial (JPPP-70). Am J Cardiovasc Drugs 2019;19(3):299-311.

20. Uchiyama S, Ishizuka N, Shimada K, et al. Aspirin for Stroke Prevention in Elderly Patients With Vascular Risk Factors: Japanese Primary Prevention Project. Stroke 2016;47(6):605-11.

21. Meinshausen M, Rieckert A, Renom-Guiteras A, et al. Effectiveness and patient safety of platelet aggregation inhibitors in the prevention of cardiovascular disease and ischemic stroke in older adults - a systematic review. BMC Geriatr 2017;17(Suppl 1):225.

22. Li L, Geraghty OC, Mehta Z, Rothwell PM; Oxford Vascular Study. Age-specific risks, severity, time course, and outcome of bleeding on long-term antiplatelet treatment after vascular events: a population-based cohort study. Lancet 2017;390(10093):490-99.

23. Di Bari M, Degli Esposti L, Veronesi C, et al. Combination evidence-based therapy is effective in the oldest 'old patients' following myocardial infarction. The "Salute e Benessere nell'Anziano" (SeBA) observational study. Intern Emerg Med 2016;11(5):677-85.

24. Di Nora C, Cioffi G, Iorio A, et al. Systolic blood pressure target in systemic arterial hypertension: Is lower ever better? Results from a community-based Caucasian cohort. Eur J Intern Med 2018;48:57-63.

25. Dregan A, Ravindrarajah R, Hazra N, Hamada S, Jackson SH, Gulliford MC. Longitudinal Trends in Hypertension Management and Mortality Among Octogenarians: Prospective Cohort Study. Hypertension 2016;68(1):97-105.

26. Streit S, Poortvliet RKE, Gussekloo J. Lower blood pressure during antihypertensive treatment is associated with higher all-cause mortality and accelerated cognitive decline in the oldest-old-data from the Leiden 85-plus Study. Age Ageing 2018;1;47(4):545-50.

27. van Dalen JW, Moll van Charante EP, van Gool WA, Richard E. Discontinuation of Antihypertensive Medication, Cognitive Complaints, and Incident Dementia. J Am Med Dir Assoc 2019;20(9):1091-97.e3.

28. McNeil JJ, Wolfe R, Woods RL et al. Effect of Aspirin on Cardiovascular Events and Bleeding in the Healthy Elderly. N Engl J Med 2018;379(16):1509-18.

29. McNeil JJ, Woods RL, Nelson MR, et al. Baseline Characteristics of Participants in the ASPREE (ASPirin in Reducing Events in the Elderly) Study. J Gerontol A Biol Sci Med Sci 2017;72(11):1586-93.

30. Kirk JK, Allsbrook J, Hansell M, Mann EM. A systematic review of hypertension outcomes and treatment strategies in older adults. Arch Gerontol Geriatr 2017;73:160-68.

31. Mühlbauer V, Dallmeier D, Brefka S, Bollig C, Voigt-Radloff S, Denkinger M. The Pharmacological Treatment of Arterial Hypertension in Frail, Older Patients-a Systematic Review. Dtsch Arztebl Int 2019;116(3):23-30.

32. Zhang XE, Cheng B, Wang Q. Relationship between high blood pressure and cardiovascular outcomes in elderly frail patients: A systematic review and meta-analysis. Geriatr Nurs 2016; 37(5):385-92.

33. Burton JK, Papworth R, Haig C, et al. Statin Use is Not Associated with Future Long-Term Care Admission: Extended Follow-Up of Two Randomised Controlled Trials. Drugs Aging 2018;35(7):657-63.

34. Cheung BMY, Lam KSL. Never too old for statin treatment? Lancet 2019;393(10170):379-80.

35. Benetos A, Labat C, Rossignol P, et al. Treatment With Multiple Blood Pressure Medications, Achieved Blood Pressure, and Mortality in Older Nursing Home Residents: The PARTAGE Study. JAMA Intern Med 2015;175(6):989-95.

36. Streit S, Poortvliet RKE, Elzen WPJD, Blom JW, Gussekloo J. Systolic Blood Pressure and Cognitive Decline in Older Adults With Hypertension. Ann Fam Med 2019;17(2):100-7.

37. Dewhurst F, Baker L, Andrew I, Todd A. Blood pressure evaluation and review of antihypertensive medication in patients with life limiting illness. Int J Clin Pharm 2016; 38(5):1044-7.

38. Gulla C, Flo E, Kjome RL, Husebo BS. Deprescribing antihypertensive treatment in nursing home patients and the effect on blood pressure. J Geriatr Cardiol 2018;15(4):275-83.

39. McNeil JJ, Woods RL, Nelson MR, et al. Effect of Aspirin on Disability-free Survival in the Healthy Elderly. N Engl J Med 2018;379(16):1499-1508.

40. McNeil JJ, Nelson MR, Woods RL, et al., Effect of Aspirin on All-Cause Mortality in the Healthy Elderly. N Engl J Med 2018;379(16):1519-28.

41. Han BH, Sutin D, Williamson JD, et al. Effect of Statin Treatment vs Usual Care on Primary Cardiovascular Prevention Among Older Adults: The ALLHAT-LLT Randomized Clinical Trial. JAMA Intern Med 2017;177(7):955-65.

## Optional literature

1. Alter DA, Tu JV, Koh M, et al. Projected Real-World Effectiveness of Using Aggressive Low-Density Lipoprotein Cholesterol Targets Among Elderly Statin Users Following Acute Coronary Syndromes in Canada. J Am Heart Assoc 2018;12;7(10):e007535.

2. Chiu HT, Shen LJ, Chen YC, Lin JH, Wang CC. Effect of statin use on the risk of medically attended acute respiratory illness among influenza vaccinated elderly. Vaccine 2018;36(41):6133-37.

3. Jones M, Tett S, Peeters GM, Mishra GD, Dobson A. New-Onset Diabetes After Statin Exposure in Elderly Women: The Australian Longitudinal Study on Women's Health. Drugs Aging 2017;34(3):203-9.

4. Kwak A, Kim JH, Choi CU, Kim IW, Oh JM, Kim K. Comparative effectiveness of statins in secondary prevention among the older people aged 75 years and over. Int J Clin Pharm 2019;41(2):460-69.

5. Noaman S, Al-Mukhtar O, Abramovic S, et al. Changes in Statin Prescription Patterns in Patients Admitted to an Australian Geriatric Subacute Unit. Heart Lung Circ 2019;28(3): 423-29.

6. Rea F, Calusi G, Franchi M, et al. Adherence of Elderly Patients with Cardiovascular Disease to Statins and the Risk of Exacerbation of Chronic Obstructive Pulmonary Disease: Evidence from an Italian Real-World Investigation. Drugs Aging 2018;35(12):1099-1108.

7. Spannella F, Giulietti F, Balietti P, et al. Renin-Angiotensin System Blockers and Statins Are Associated With Lower In-Hospital Mortality in Very Elderly Hypertensives. J Am Med Dir Assoc 2018;19(4):342-47.

8. Leya M, Stone NJ. Statin. Statin Prescribing in the Elderly: Special Considerations. Curr Atheroscler Rep 2017;19(11):47.

9. Bergström H, Brånvall E, Helde-Frankling M, Björkhem-Bergman L. Differences in discontinuation of statin treatment in women and men with advanced cancer disease. Biol Sex Differ 2018;9(1):47.

10. Gulliford M, Ravindrarajah R, Hamada S, Jackson S, Charlton J. Inception and deprescribing of statins in people aged over 80 years: cohort study. Age Ageing 2017;46(6):1001-5.

11. McNeil MJ, Kamal AH, Kutner JS, Ritchie CS, Abernethy AP. The Burden of Polypharmacy in Patients Near the End of Life. J Pain Symptom Manage 2016;51(2):178-83.e2.

12. Streit S, Verschoor M, Rodondi N et al. Variation in GP decisions on antihypertensive treatment in oldest-old and frail individuals across 29 countries. BMC Geriatr 2017;17(1):93.

13. Zullo AR, Olean M, Berry SD, Lee Y, Tjia J, Steinman MA. Patient-Important Adverse Events of beta-blockers in Frail Older Adults after Acute Myocardial Infarction. J Gerontol A Biol Sci Med Sci 2019;74(8):1277-81.

14. Weiss J, Freeman M, Low A, Fu R, et al. Benefits and Harms of Intensive Blood Pressure Treatment in Adults Aged 60 Years or Older: A Systematic Review and Meta-analysis. Ann Intern Med 2017;166(6):419-29.

15. Baena Díez JM, López Maldonado N, Navarro Guiu E, Alcayde Claveria D, García Lareo M, Pérez Orcero A. Antihypertensive overtreatment in people 80 years old and older. Med Clin (Barc) 2018;150(6):220-23.

16. Rocca B, Husted S. Safety of Antithrombotic Agents in Elderly Patients with Acute Coronary Syndromes. Drugs Aging 2016;33(4):233-48.

17. Sehgal M, Wood SK, Ouslander JG, Hennekens CH. Aspirin in Older Adults: Need for Wider Utilization in Secondary Prevention and Individual Clinical Judgments in Primary Prevention. J Cardiovasc Pharmacol Ther 2017;22(6):511-13.

18. Zheng SL, Roddick AJ.Association of Aspirin Use for Primary Prevention With Cardiovascular Events and Bleeding Events: A Systematic Review and Meta-analysis. JAMA 2019;321(3):277-87.

19. Blin P, Dureau-Pournin C, Lassalle R, et al. Outcomes in patients after myocardial infarction similar to those of the PEGASUS-TIMI 54 trial: A cohort study in the French national claims database. Br J Clin Pharmacol 2017;83(9):2056-65.

20. Upadhaya S, Madala S, Baniya R, Saginala K, Khan J. Impact of acetylsalicylic acid on primary prevention of cardiovascular diseases: A meta-analysis of randomized trials. Eur J Prev Cardiol 2019;26(7):746-749.

21. Benetos A, Rossignol P, Cherubini A, et al. Polypharmacy in the Aging Patient: Management of Hypertension in Octogenarians. JAMA 2015;314(2):170-80.

22. Mossello E, Pieraccioli M, Nesti N, et al. Effects of low blood pressure in cognitively impaired elderly patients treated with antihypertensive drugs. JAMA Intern Med 2015;175(4):578-85.

23. Ridker PM. Should Aspirin Be Used for Primary Prevention in the Post-Statin Era? N Engl J Med 2018;379(16):1572-74

24. Gaziano JM. Aspirin for Primary Prevention: Clinical Considerations in 2019. JAMA 2019;22;321(3):253-55.
