# Supplementary material for: Assessment of the appropriateness of cardiovascular preventive medication in older people: using the RAND/UCLA Appropriateness Method
Source: BMC Geriatr. 2022 May 5;22:394. doi: 10.1186/s12877-022-03082-8 (PMC9069851; doi:10.1186/s12877-022-03082-8)
Supplement: Supplementary file 2 — Additional file 2. Construction of the rating sheets: detailed information. This file includes a detailed description of how the rating sheets were constructed, and an example of a rating sheet. [file 12877_2022_3082_MOESM2_ESM.doc]

# **Additional file 2**

# **Construction of the rating sheets: detailed information**

## Rationale behind the variable categories

The rating sheets were constructed based on combinations of a set of variables. Each combination of variables was considered a (theoretical) clinical scenario. When life expectancy was <1 years, some of the categories were collapsed because we assumed that the appropriateness of treatment would be similar for the different categories. Also, because we considered it unlikely to have a life expectancy of < 1 year without experiencing any health problems, we excluded the category of 0 domains of the variable ‘*Complexity of health problems’*. To create enough contrast between the systolic blood pressure (SBP) values, we choose categories of 20mmHg difference. In the clinical scenarios about platelet aggregation inhibitors (PAI) we made a distinction between mild and severe hindering side effects. This distinction was not made for cholesterol lowering medication or antihypertensives. The difference between mild and severe hindering side effects of PAI was considered to be most relevant for daily practice. Dividing the hindering side effects of antihypertensives and cholesterol lowering medication into mild and severe would result in a substantial increase in the already high number of rating sheets. The addition of more rating sheets was not feasible.

In Table 2.1 the variables and the categories of the variables are listed stratified by life expectancy.

**Table 2.1 Categories of the different clinical variables used in the rating sheets**

| **Clinical variables** | **Normal life expectancy** | **Life expectancy < 1year** |
| --- | --- | --- |
| **Complexity of health problems**  (number of domains with problems) | 0;1;2;3-4 | 1-2; 3-4 |
| **Age (yrs)** | 75 -85; >85 | ≥ 75 |
| **Side effects^1^** | absence; present^2^ | absence; present |
| **Cardiovascular event** | none; < 1year ago; ≥ 1 year ago | none; < 1year ago; ≥ 1 year ago |
| **SBP level (mmHg)** | 120; 140;160;180 | 120;140;160;180 |
| **LDL-C level mmol/l** | ≤2.5 and > 2.5 | ≤2.5 and > 2.5 |

^1^Side effects were only included in the clinical scenarios about stopping treatment.

^2^side effects of platelet aggregation inhibitors were judged separately for mild (medical attention not required) and severe (medical attention required) side effects.

## Assumptions

Several assumptions were made to prevent the creation of unlikely clinical scenarios and to limit the number of clinical scenarios. These assumptions were presented to the panel in round 1 and differences were discussed during the clarifying session during the face-to-face meeting (Table 2.2). As result we added extra rating sheets to round 2, to assess the appropriateness of starting antihypertensive treatment when SBP=>180mmHg.

Since it is generally not recommended by the Dutch guidelines on cardiovascular disease prevention to start cholesterol lowering treatment in adults >75 years when LDL-C ≤2.5 mmol/l, nor to start antihypertensive treatment when SBP=120mmHg, these clinical scenarios were not included in the rating sheets^1^.

**Table 2.2 List of assumptions round 1**

| **Assumption** |
| --- |
| - It’s extremely appropriate to stop cholesterol lowering treatment in adults >75 years with hindering side effects when LDL-C ≤2.5 mmol/l - It’s extremely appropriate to stop antihypertensives when SBP ≤120 mmHg in presence of hindering side effects, independent from age, number of domains with health problems, and life expectancy - It’s extremely appropriate to start antihypertensives when SBP ≥180 mmHg - It’s extremely inappropriate to stop antihypertensives when SBP ≥180 mmHg - The appropriateness of stopping CPM is independent of age when life expectancy is < 1 year. |

**Reference**

1. Dutch College of General Practitioners; Cardiovascular Risk Managment (CVRM) 2019. Available from: [https://richtlijnendatabase.nl/richtlijn/cardiovasculair_risicomanagement_cvrm/inleiding_kwetsbaarheid.html#tab-content-accountability](https://richtlijnendatabase.nl/richtlijn/cardiovasculair_risicomanagement_cvrm/inleiding_kwetsbaarheid.html#tab-content-accountability) accessed 05-05-2020

## Example of a rating sheet of round 1

**STARTING antihypertensive treatment, no history of cardiovascular disease, life expectancy ≥ 1 year, SBP = 140mmHg**

Panelist:

Appropriateness scale: 1= extremely inappropriate to 9= extremely appropriate

How appropriate do you consider **starting antihypertensive treatment** in an older adult with a **SBP =140 mmHg** and a **life expectancy ≥ 1 year**?

**Instruction: please encircle your answer.**

| **No ASCVD Score round 2** | | | | |
| --- | --- | --- | --- | --- |
| **SBP =140 mmHg** | **0 domains** | **1 domains** | **2 domains** | **3-4 domains** |
| **75 to 85 years** | 1 2 3 4 5 6 7 8 9 | 1 2 3 4 5 6 7 8 9 | 1 2 3 4 5 6 7 8 9 | 1 2 3 4 5 6 7 8 9 |
| **> 85 years** | 1 2 3 4 5 6 7 8 9 | 1 2 3 4 5 6 7 8 9 | 1 2 3 4 5 6 7 8 9 | 1 2 3 4 5 6 7 8 9 |
