# Supplementary material for: Assessment of the appropriateness of cardiovascular preventive medication in older people: using the RAND/UCLA Appropriateness Method
Source: BMC Geriatr. 2022 May 5;22:394. doi: 10.1186/s12877-022-03082-8 (PMC9069851; doi:10.1186/s12877-022-03082-8)
Supplement: Supplementary file 3 — Additional file 3. Additional considerations mentioned in the discussions. This text file contains additional considerations that were discussed regarding the appropriateness of platelet aggregation inhibitors and cholesterol lowering medication in older adults. [file 12877_2022_3082_MOESM3_ESM.doc]

# **Additional file 3**

# **Additional considerations mentioned in the discussions**

## The influence of a history of atherosclerotic cardiovascular disease (ASCVD) on Platelet aggregation inhibitors (PAI) and cholesterol lowering medication

They panellists discussed that in absence of ASCVD, not only the expected benefit of PAI is low, but PAI can also be harmful. Therefore, they judged for all clinical scenarios without ASCVD it was inappropriate to start and appropriate to stop PAI. Also, for cholesterol lowering medication, it was judged inappropriate to start, and mostly judged uncertain or appropriate to stop when LDL-C >2.5 mmol/L. The main arguments mentioned in regard to cholesterol lowering treatment were, the limited evidence in support of the benefits of cholesterol reduction in older adults without ASCVD, and a high number needed to treat. The importance of supporting scientific evidence was also emphasized by the older representatives.

## The severity of side effects

The panelists reasoned that mild side effect of PAI generally have a limited impact on quality of life, and judged that PAI remain appropriate for most older adults with a history of ASCVD. Although, it was said that the burden of mild side effects may be heavier when health problems are complex. When side effects were severe, the panelists were uncertain about the appropriateness of PAI for the relatively healthy, as these side effects are mostly transient and often don’t re-occur. In the remaining clinical scenarios with severe side effects, regarding those with more complex health problems, PAI were judged inappropriate. This last was much debated; on the one hand it was discussed that for this group the risk of recurrence of a bleeding is higher, and the prognosis of a serious bleeding complication is worse. On the other hand, the risk of a recurrent ASCVD-event is also high.
