# Supplementary material for: Assessment of the appropriateness of cardiovascular preventive medication in older people: using the RAND/UCLA Appropriateness Method
Source: BMC Geriatr. 2022 May 5;22:394. doi: 10.1186/s12877-022-03082-8 (PMC9069851; doi:10.1186/s12877-022-03082-8)
Supplement: Supplementary file 4 — Additional file 4. Appropriateness scores of starting and stopping cardiovascular preventive medication. This file includes twelve tables (3.1–3.12) that display the appropriateness scores (panel medians) of rating round 2. [file 12877_2022_3082_MOESM4_ESM.doc]

# Additional file 4

# Appropriateness scores of starting and stopping cardiovascular preventive medication

The tables in this file provide additional information to Figure 3-5 of the main article. Tables 3.1-3.3 correspond with Figure 3; Tables 3.4-3.6 with Figure 4 and Tables 3.7-3.9 with Figure 5. The data in Tables 3.10-3.12 is not shown in the main article.

In round two there was disagreement on 3 of the total of 474 clinical scenarios.

**Abbreviations:**

**ASCVD:** atherosclerotic cardiovascular disease

**D**=Disagreement: at least four panelist rated in the 1-3 range and at least four panelists rated in the 7-9 range.

**LDL-C**= Low-density lipoprotein cholesterol

**SBP**= systolic blood pressure.

**n.a.** not applicable. These scenarios were not included in the rating sheets, see the assumptions presented in Table 2.2 of Additional file 2.

**Table 3.1.** Panel medians of the appropriateness score of starting and stopping cardiovascular preventive medication for clinical scenarios without a history of ASCVD (normal life expectancy and no side effects)

| **No history of ASCVD** |  | **Panel medians of the appropriateness score of starting** | | | | |  | **Panel medians of the appropriateness score of stopping** | | | | | |
| --- | --- | --- | --- | --- | --- | --- | --- | --- | --- | --- | --- | --- | --- |
|  |  | **Health domains with problems** | | | | |  | **Health domains with problems** | | | | | |
|  | **Age** | 0 | 1 | 2 | 3-4 |  | | | 0 | 1 | 2 | 3-4 |  |
| **Cholesterol lowering medication** |  |  |  |  |  |  | | |  |  |  |  |  |
| LDL-C ≤ 2.5 mmol/l | >85 | n.a. | n.a. | n.a. | n.a. |  | | | 6.5 | 7 | 8.5 | 9 |  |
|  | 75-85 | n.a. | n.a. | n.a. | n.a. |  | | | D | D | 7 | 8 |  |
| LDL-C >2.5 mmol/l | >85 | 1 | 1 | 1 | 1 |  | | | 5.5 | 6.5 | 8 | 8.5 |  |
|  | 75-85 | 3 | 2.5 | 1.5 | 1 |  | | | 3 | 4 | 6 | 7 |  |
| **Platelet aggregation inhibitors** |  |  |  |  |  |  | | |  |  |  |  |  |
|  | >85 | 1 | 1 | 1 | 1 |  | | | 9 | 9 | 9 | 9 |  |
|  | 75-85 | 1 | 1 | 1 | 1 |  | | | 9 | 9 | 9 | 9 |  |
| **Antihypertensive medication** |  |  |  |  |  |  | | |  |  |  |  |  |
| SBP 120 mmHg | >85 | n.a. | n.a. | n.a. | n.a. |  | | | 7 | 7 | 7 | 8 |  |
|  | 75-85 | n.a. | n.a. | n.a. | n.a. |  | | | 6 | 6 | 7 | 8 |  |
| SBP 140 mmHg | >85 | 2 | 2 | 1 | 1 |  | | | 4.5 | 5.5 | 6 | 6.5 |  |
|  | 75-85 | 2 | 2 | 1 | 1 |  | | | 3 | 3.5 | 4.5 | 6 |  |
| SBP 160 mmHg | >85 | 6 | 5 | 4 | 3.5 |  | | | 3 | 3.5 | 4.5 | 5 |  |
|  | 75-85 | 7 | 7 | 5.5 | 4.5 |  | | | 2 | 2.5 | 3.5 | 4.5 |  |
| SBP 180 mmHg | >85 | 7 | 7 | 5 | D |  | | | n.a. | n.a. | n.a. | n.a. |  |
|  | 75-85 | 8.5 | 8 | 7 | 6.5 |  | | | n.a. | n.a. | n.a. | n.a. |  |

**Table 3.2.** Panel medians of the appropriateness score of starting and stopping cardiovascular preventive medication for clinical scenarios with a cardiovascular event ≥1 year (normal life expectancy and no side effects)

| **ASCVD ≥1 year** |  | **Panel medians of the appropriateness score of starting** | | | |  | **Panel medians of the appropriateness score of stopping** | | | |
| --- | --- | --- | --- | --- | --- | --- | --- | --- | --- | --- |
|  |  |  | | | |  |  | | | |
|  |  | **Health domains with problems** | | | |  | **Health domains with problems** | | | |
|  | **Age** | **0** | **1** | **2** | **3-4** |  | **0** | **1** | **2** | **3-4** |
| **Cholesterol lowering medication** |  |  |  |  |  |  |  |  |  |  |
| LDL-C ≤ 2.5 mmol/l | >85 | n.a. | n.a. | n.a. | n.a. |  | 5 | 5 | 5.5 | 7 |
|  | 75-85 | n.a. | n.a. | n.a. | n.a. |  | 4 | 4 | 5 | 5.5 |
| LDL-C >2.5 mmol/l | >85 | 5 | 4.5 | 3.5 | 2 |  | 4 | 4 | 5.5 | 7 |
|  | 75-85 | 6 | 6 | 5 | 3 |  | 3 | 3.5 | 5 | 5 |
| **Platelet aggregation inhibitors** |  |  |  |  |  |  |  |  |  |  |
|  | >85 | 8 | 8 | 7 | 6 |  | 2 | 3 | 3 | 4 |
|  | 75-85 | 9 | 9 | 8 | 7 |  | 2 | 2 | 3 | 4 |
| **Antihypertensive medication** |  |  |  |  |  |  |  |  |  |  |
| SBP 120 mmHg | >85 | n.a. | n.a. | n.a. | n.a. |  | 6 | 6 | 7 | 8 |
|  | 75-85 | n.a. | n.a. | n.a. | n.a. |  | 5 | 5 | 6 | 7 |
| SBP 140 mmHg | >85 | 3.5 | 3 | 2 | 1 |  | 3 | 3 | 5 | 5 |
|  | 75-85 | 4.5 | 4 | 2.5 | 2.5 |  | 2 | 2 | 4 | 5 |
| SBP 160 mmHg | >85 | 7 | 6 | 5 | 4 |  | 2 | 3 | 3 | 5 |
|  | 75-85 | 8 | 7 | 6 | 5 |  | 1 | 2 | 3 | 3 |
| SBP 180 mmHg | >85 | 8 | 7 | 7 | 6 |  | n.a. | n.a. | n.a. | n.a. |
|  | 75-85 | 8 | 8 | 7 | 7 |  | n.a. | n.a. | n.a. | n.a. |

**Table 3.3.** Panel medians of the appropriateness score of starting and stopping cardiovascular preventive medication for clinical scenarios with a cardiovascular event <1 year (normal life expectancy and no side effects)

| **ASCVD <1 year** |  | **Panel medians of the appropriateness score of starting** | | | |  | **Panel medians of the appropriateness score of stopping** | | | |
| --- | --- | --- | --- | --- | --- | --- | --- | --- | --- | --- |
|  |  | **Health domains with problems** | | | |  | **Health domains with problems** | | | |
|  | **Age** | **0** | **1** | **2** | **3-4** |  | **0** | **1** | **2** | **3-4** |
| **Cholesterol lowering medication** |  |  |  |  |  |  |  |  |  |  |
| LDL-C ≤ 2.5 mmol/l | >85 | n.a. | n.a. | n.a. | n.a. |  | 4 | 4 | 5 | 6 |
|  | 75-85 | n.a. | n.a. | n.a. | n.a. |  | 3 | 4 | 4.5 | 5 |
| LDL-C >2.5 mmol/l | >85 | 5 | 5 | 4 | 2.5 |  | 3 | 3 | 5 | 6 |
|  | 75-85 | 7 | 7 | 6 | 5 |  | 2 | 3 | 4 | 5 |
| **Platelet aggregation inhibitors** |  |  |  |  |  |  |  |  |  |  |
|  | >85 | 9 | 9 | 8 | 7 |  | 1 | 2 | 2 | 3 |
|  | 75-85 | 9 | 9 | 8 | 8 |  | 1 | 1 | 2 | 3 |
| **Antihypertensive medication** |  |  |  |  |  |  |  |  |  |  |
| SBP 120 mmHg | >85 | n.a. | n.a. | n.a. | n.a. |  | 6 | 6 | 7 | 7 |
|  | 75-85 | n.a. | n.a. | n.a. | n.a. |  | 5 | 5 | 6 | 7 |
| SBP 140 mmHg | >85 | 4.5 | 4.5 | 2.5 | 2 |  | 3 | 3 | 4 | 5 |
|  | 75-85 | 5 | 4.5 | 3.5 | 3 |  | 2 | 2 | 3 | 4 |
| SBP 160 mmHg | >85 | 7 | 6 | 6 | 5 |  | 2 | 2 | 3 | 4 |
|  | 75-85 | 8 | 8 | 6 | 6 |  | 1 | 1 | 2 | 3 |
| SBP 180 mmHg | >85 | 8 | 8 | 7 | 6 |  | n.a. | n.a. | n.a. | n.a. |
|  | 75-85 | 8 | 8 | 7 | 7 |  | n.a. | n.a. | n.a. | n.a. |

**Table 3.4.** Panel medians of the appropriateness score of stopping cardiovascular preventive medication for clinical scenarios without a history of cardiovascular disease in presence of side effects (normal life expectancy)

| **Side effects**  **No history of ASCVD** |  | **Panel medians of the appropriateness score of stopping** | | | |
| --- | --- | --- | --- | --- | --- |
|  |  | **Health domains with problems** | | | |
|  | **Age** | 0 | 1 | 2 | 3-4 |
| **Cholesterol lowering medication** |  |  |  |  |  |
| LDL-C >2.5 mmol/l | >85 | 9 | 9 | 9 | 9 |
|  | 75-85 | 8.5 | 9 | 9 | 9 |
| **Platelet aggregation inhibitors** |  |  |  |  |  |
| Mild side effects | >85 | 9 | 9 | 9 | 9 |
|  | 75-85 | 9 | 9 | 9 | 9 |
| Severe side effects | >85 | 9 | 9 | 9 | 9 |
|  | 75-85 | 9 | 9 | 9 | 9 |
| **Antihypertensive medication** |  |  |  |  |  |
| SBP 120 mmHg | >85 | n.a. | n.a. | n.a. | n.a. |
|  | 75-85 | n.a. | n.a. | n.a. | n.a. |
| SBP 140 mmHg | >85 | 8 | 8 | 8 | 9 |
|  | 75-85 | 7 | 7 | 8 | 9 |
| SBP 160 mmHg | >85 | 7 | 7 | 8 | 8.5 |
|  | 75-85 | 6 | 7 | 7 | 8 |

**Table 3.5**. Panel medians of the appropriateness score of stopping cardiovascular preventive medication for clinical scenarios with a cardiovascular event ≥1 year, in presence of side effects (normal life expectancy)

| **Side effects**  **ASCVD ≥1 year** |  |  | **Panel medians of the appropriateness score of stopping** | | | |
| --- | --- | --- | --- | --- | --- | --- |
|  |  |  | **Health domains with problems** | | | |
|  | **Age** |  | 0 | 1 | 2 | 3-4 |
| **Cholesterol lowering medication** |  |  |  |  |  |  |
| LDL-C >2.5 mmol/l | >85 |  | 8 | 8 | 9 | 9 |
|  | 75-85 |  | 6.5 | 7 | 8 | 8.5 |
| **Platelet aggregation inhibitors** |  |  |  |  |  |  |
| Mild side effects | >85 |  | 3 | 3 | 4 | 5 |
|  | 75-85 |  | 3 | 3 | 3 | 4 |
| Severe side effects | >85 |  | 6 | 7 | 7 | 8 |
|  | 75-85 |  | 5 | 6 | 7 | 8 |
| **Antihypertensive medication** |  |  |  |  |  |  |
| SBP 120 mmHg | >85 |  | n.a. | n.a. | n.a. | n.a. |
|  | 75-85 |  | n.a. | n.a. | n.a. | n.a. |
| SBP 140 mmHg | >85 |  | 7 | 7 | 8 | 8 |
|  | 75-85 |  | 6 | 6 | 7 | 8 |
| SBP 160 mmHg | >85 |  | 5 | 6 | 7 | 8 |
|  | 75-85 |  | 4 | 5 | 6 | 7 |

**Table 3.6.** Panel medians of the appropriateness score of stopping cardiovascular preventive medication for clinical scenarios with a cardiovascular event <1 year ago, in presence of side effects (normal life expectancy)

| **Side effects**  **ASCVD <1 year** |  |  | **Panel medians of the appropriateness score of stopping** | | | |
| --- | --- | --- | --- | --- | --- | --- |
|  |  |  | **Health domains with problems** | | | |
|  | **Age** |  | 0 | 1 | 2 | 3-4 |
| **Cholesterol lowering medication** |  |  |  |  |  |  |
| LDL-C >2.5 mmol/l | >85 |  | 7 | 7.5 | 9 | 9 |
|  | 75-85 |  | 6 | 7 | 8 | 8.5 |
| **Platelet aggregation inhibitors** |  |  |  |  |  |  |
| Mild side effects | >85 |  | 2 | 3 | 3 | 4 |
|  | 75-85 |  | 2 | 2 | 3 | 3 |
| Severe side effects | >85 |  | 6 | 6 | 7 | 8 |
|  | 75-85 |  | 5 | 6 | 7 | 7 |
| **Antihypertensive medication** |  |  |  |  |  |  |
| SBP 120 mmHg | >85 |  | n.a. | n.a. | n.a. | n.a. |
|  | 75-85 |  | n.a. | n.a. | n.a. | n.a. |
| SBP 140 mmHg | >85 |  | 6 | 6 | 8 | 9 |
|  | 75-85 |  | 5 | 6 | 7 | 8 |
| SBP 160 mmHg | >85 |  | 5 | 5 | 6 | 7 |
|  | 75-85 |  | 4 | 5 | 5 | 6 |

**Table 3.7.** Panel medians of the appropriateness score of starting and stopping cardiovascular preventive medication for clinical scenarios without a history of ASCVD when life expectancy <1 year (no side effects)

| **Life expectancy <1 year** |  | **Panel medians of the appropriateness score of starting** | |  | **Panel medians of the appropriateness score of stopping** | |
| --- | --- | --- | --- | --- | --- | --- |
| **No history of ASCVD** |  | **Health domains with problems** | |  | **Health domains with problems** | |
|  | **Age** | **1-2** | **3-4** |  | **1-2** | **3-4** |
| **Cholesterol lowering medication** |  |  |  |  |  |  |
| LDL-C ≤2.5 mmol/l | ≥75 | n.a. | n.a. |  | 9 | 9 |
| LDL-C >2.5 mmol/l | ≥75 | 1 | 1 |  | 9 | 9 |
| **Platelet aggregation inhibitors** |  |  |  |  |  |  |
|  | ≥75 | 1 | 1 |  | 9 | 9 |
| **Antihypertensive medication** |  |  |  |  |  |  |
| SBP 120 mmHg | ≥75 | n.a. | n.a. |  | 8 | 8 |
| SBP 140 mmHg | ≥75 | 1 | 1 |  | 6 | 7 |
| SBP 160 mmHg | ≥75 | 2 | 1 |  | 5 | 6 |

**Table 3.8.** Panel medians of the appropriateness score of starting and stopping cardiovascular preventive medication for clinical scenarios with a cardiovascular event ≥1 year, when life expectancy < 1 year (no side effects)

| **Life expectancy <1 year** |  | **Panel medians of the appropriateness score of starting** | |  | **Panel medians of the appropriateness score of stopping** | |
| --- | --- | --- | --- | --- | --- | --- |
| **ASCVD** ≥**1 year** |  | **Health domains with problems** | |  | **Health domains with problems** | |
|  | **Age** | **1-2** | **3-4** |  | **1-2** | **3-4** |
| **Cholesterol lowering medication** |  |  |  |  |  |  |
| LDL-C ≤2.5 mmol/l | ≥75 | n.a. | n.a. |  | 8 | 8 |
| LDL-C >2.5 mmol/l | ≥75 | 1 | 1 |  | 8 | 8.5 |
| **Platelet aggregation inhibitors** |  |  |  |  |  |  |
|  | ≥75 | 3 | 2 |  | 6 | 7 |
| **Antihypertensive medication** |  |  |  |  |  |  |
| SBP 120 mmHg | ≥75 | n.a. | n.a. |  | 8 | 8 |
| SBP 140 mmHg | ≥75 | 1 | 1 |  | 5 | 6 |
| SBP 160 mmHg | ≥75 | 2 | 2 |  | 4 | 5 |

**Table 3.9.** Panel medians of the appropriateness score of starting and stopping cardiovascular preventive medication for clinical scenarios with a cardiovascular event <1 year, when life expectancy <1 year (no side effects)

| **Life expectancy <1 year** |  | **Panel medians of the appropriateness score of starting** | |  | **Panel medians of the appropriateness score of stopping** | |
| --- | --- | --- | --- | --- | --- | --- |
| **ASCVD <1 year** |  | **Health domains with problems** | |  | **Health domains with problems** | |
|  | **Age** | **1-2** | **3-4** |  | **1-2** | **3-4** |
| **Cholesterol lowering medication** |  |  |  |  |  |  |
| LDL-C ≤2.5 mmol/l | ≥75 | n.a. | n.a. |  | 7 | 8 |
| LDL-C >2.5 mmol/l | ≥75 | 1 | 1 |  | 7 | 8 |
| **Platelet aggregation inhibitors** |  |  |  |  |  |  |
|  | ≥75 | 5 | 3 |  | 5 | 6 |
| **Antihypertensives** |  |  |  |  |  |  |
| SBP 120 mmHg | ≥75 | n.a. | n.a. |  | 7 | 7 |
| SBP 140 mmHg | ≥75 | 2 | 1 |  | 5 | 6 |
| SBP 160 mmHg | ≥75 | 2 | 2 |  | 4 | 5 |

**Table 3.10.** Panel medians of the appropriateness score of stopping cardiovascular preventive medication for clinical scenarios without a history of ASCVD in presence of side effects and life expectancy <1 year

| **Life expectancy <1 year**  **Side effects** |  |  | **Panel medians of the appropriateness score of stopping** | |
| --- | --- | --- | --- | --- |
| **No history of ASCVD** |  |  | **Health domains with problems** | |
|  | **Age** |  | **1-2** | **3-4** |
| **Cholesterol lowering medication** |  |  |  |  |
| LDL-C ≤2.5 mmol/l | ≥75 |  | n.a. | n.a. |
| LDL-C >2.5 mmol/l | ≥75 |  | 9 | 9 |
| **Platelet aggregation inhibitors** |  |  |  |  |
| Mild side effects | ≥75 |  | 9 | 9 |
| Severe side effects | ≥75 |  | 9 | 9 |
| **Antihypertensives** |  |  |  |  |
| SBP 120 mmHg | >75 |  | n.a. | n.a. |
| SBP 140 mmHg | ≥75 |  | 9 | 9 |
| SBP 160 mmHg | ≥75 |  | 8 | 8 |

**Table 3.11.** Panel medians of the appropriateness score of stopping cardiovascular preventive medication for clinical scenarios with a cardiovascular event ≥1 year in presence of side effects and life expectancy < 1 year

| **Life expectancy <1 year**  **Side effects** |  |  | **Panel medians of the appropriateness score of stopping** | |
| --- | --- | --- | --- | --- |
| **ASCVD ≥1 year** |  |  | **Health domains with problems** | |
|  | **Age** |  | **1-2** | **3-4** |
| **Cholesterol lowering medication** |  |  |  |  |
| LDL-C ≤2.5 mmol/l | ≥75 |  | n.a. | n.a. |
| LDL-C >2.5 mmol/l | ≥75 |  | 9 | 9 |
|  |  |  |  |  |
| **Platelet aggregation inhibitors** |  |  |  |  |
| Mild side effects | ≥75 |  | 5 | 6 |
| Severe side effects | ≥75 |  | 9 | 9 |
| **Antihypertensives** |  |  |  |  |
| SBP 120 mmHg | >75 |  | n.a. | n.a. |
| SBP 140 mmHg | ≥75 |  | 8 | 9 |
| SBP 160 mmHg | ≥75 |  | 7 | 9 |

**Table 3.12.** Panel medians of the appropriateness score of stopping cardiovascular preventive medication for clinical scenarios with a cardiovascular event <1 year in presence of side effects and life expectancy < 1 year

| **Life expectancy <1 year**  **Side effects** |  |  | **Panel medians of the appropriateness score of stopping** | |
| --- | --- | --- | --- | --- |
| **ASCVD <1 year** |  |  | **Health domains with problems** | |
|  | **Age** |  | **1-2** | **3-4** |
| **Cholesterol lowering medication** |  |  |  |  |
| LDL-C ≤2.5 mmol/l | ≥75 |  | n.a. | n.a. |
| LDL-C >2.5 mmol/l | ≥75 |  | 9 | 9 |
| **Platelet aggregation inhibitors** |  |  |  |  |
| Mild side effects | ≥75 |  | 5 | 5 |
| Severe side effects | ≥75 |  | 8 | 9 |
| **Antihypertensives** |  |  |  |  |
| SBP 120 mmHg | >75 |  | n.a. | n.a. |
| SBP 140 mmHg | ≥75 |  | 8 | 8 |
| SBP 160 mmHg | ≥75 |  | 7 | 7 |
